# Supplementary material for: Early COVID-19 Interventions Failed to Replicate 1918 St. Louis vs. Philadelphia Outcomes in the United States
Source: Front Public Health. 2020 Sep 15;8:579559. doi: 10.3389/fpubh.2020.579559 (PMC7522277; doi:10.3389/fpubh.2020.579559)
Supplement: Supplementary file 1 [file Table_1.PDF]

Supplemental Table 1. Stay at home orders put in place in each state in the United States.

US State	Description	State Date (2020)[1,2,3]	End Date (2020)[1,2,3]	Duration (Days)	Cases at Start[4]	Cases at End Date
Alabama	Stay at home	April 4	April 30	27	1495	7088
Alaska	Shelter in place	March 28	April 21	25	58	329
Arizona	Stay home, stay healthy, stay connected	March 31	May 15	46	1157	13169
Arkansas[5]	-	-	-	-	-	-
California	Shelter in place	March 19	May 7	50	828	62148
Colorado	Stay at home	March 26	April 26	32	1069	13269
Connecticut	Stay safe, stay home	March 23	May 20	59	327	38815
Delaware	Stay at home	March 24	May 31	69	68	9447
District of Columbia[6]	Stay at home	April 1	June 8	69	495	9389
Florida	Stay at home	April 3	April 29	27	8999	33186
Georgia	Shelter in place	April 3	April 30	28	4570	24908
Hawaii	Stay at home	March 25	May 31	68	70	642
Idaho	Stay home	March 25	April 30	37	81	1984
Illinois	Stay at home	March 21	May 31	72	418	120057
Indiana	Stay at home	March 24	May 4	42	214	20507
Iowa[5]	-	-	-	-	-	-
Kansas	Stay home	March 30	May 3	35	330	5156
Kentucky	Stay healthy at home	March 26	May 10	46	143	6406
Louisiana	Stay at home	March 23	May 15	54	787	33772

US State	Description	State Date (2020)[1,2,3]	End Date (2020)[1,2,3]	Duration (Days)	Cases at Start[4]	Cases at End Date
Maine	Stay at home	April 2	May 31	60	297	2323
Maryland	Stay at home	March 30	May 15	47	1239	36986
Massachusetts	Stay at home	March 24	May 18	56	723	89174
Michigan	Stay home, stay safe	March 24	May 28	66	1450	57193
Minnesota	Stay at home	March 27	May 17	52	344	15639
Mississippi	Shelter in place	April 3	April 27	25	1177	6094
Missouri	Stay home Missouri	April 6	May 3	28	2393	8103
Montana	Stay at home	March 28	April 26	30	109	448
Nebraska[5]	-	-	-	-	-	-
Nevada	Stay at home	April 1	May 15	45	1012	6733
New Hampshire	Stay at home	March 27	June 15	81	137	5340
New Jersey	Stay at home	March 21	June 9	81	798	164541
New Mexico	Stay at home	March 24	May 31	69	83	7462
New York	New York State on PAUSE	March 22	May 28	68	11727	366733
North Carolina	Stay at home	March 30	May 8	40	1191	14006
North Dakota[5]	-	-	-	-	-	-
Ohio	Stay at home	March 23	May 29	68	356	34566
Oklahoma[7]	Safer at home	March 24	May 24	62	81	6037
Oregon	Stay at home	March 23	May 15	54	161	3541

US State	Description	State Date (2020)[1,2,3]	End Date (2020)[1,2,3]	Duration (Days)	Cases at Start[4]	Cases at End Date
Pennsylvania	Stay at home	March 30	June 4	67	3432	78335
Rhode Island	Stay at home	March 28	May 8	42	132	8519
South Carolina	Home or work	April 6	May 4	29	2049	6757
South Dakota[5]	-	-	-	-	-	-
Tennessee	Safer at home	March 31	April 30	31	1570	10448
Texas	Shelter in place	April 2	April 30	29	4355	28727
Utah	Stay Safe, stay home	March 27	May 1	36	368	4455
Vermont	Stay at home	March 25	May 15	52	95	929
Virginia	Stay at home	March 30	June 10	73	890	52177
Washington	Stay at home	March 23	May 31	70	1833	21634
West Virginia	Stay at home	March 24	May 3	41	16	1195
Wisconsin	Safer at home	March 25	May 13	50	480	10898
Wyoming[5]	-	-	-	-	-	-

[1] Stay at home orders start and end dates as listed on executive orders issued by each respective state.
[2] Chart: Each State's Stay-at-Home Orders and Reopening Dates, June 23, 2020, National Academy for State Health Policy.
[3] Stay on Top of "Stay At Home" – A List of Statewide Orders, May 20, 2020, Littler Mendelson, Littler.
[4] The number of cumulative confirmed COVID-19 cases in each state on the day before the start date of their stay at home order.
[5] Six states – Arkansas, Iowa, Nebraska, North Dakota, South Dakota, and Wyoming did not issue statewide stay at home orders.
[6] District of Columbia is the capital city of the United States and is not a state.
[7] The stay at home order was limited to the elderly and vulnerable populations only.
