# Supplementary material for: Early COVID-19 Interventions Failed to Replicate 1918 St. Louis vs. Philadelphia Outcomes in the United States
Source: Front Public Health. 2020 Sep 15;8:579559. doi: 10.3389/fpubh.2020.579559 (PMC7522277; doi:10.3389/fpubh.2020.579559)
Supplement: Supplementary file 2 [file Table_2.PDF]

Supplemental Table 2. New York state-level public health response to COVID-19 pandemic.

Date	New York State Response to COVID-19
3/3/20	Governor Cuomo signs $40 million Emergency Management Authorization for Coronavirus response.
3/4/20	Governor Cuomo announces SUNY and CUNY Study abroad programs in China, Italy, Japan, Iran, and South Korea are suspended immediately.
3/7/20	Governor Cuomo declares a State of Emergency to control the spread of Coronavirus.
3/9/20	Governor Cuomo announces the state will provide alcohol-based hand sanitizer to New Yorkers free of charge.
3/12/20	Governor Cuomo announces mass gathering restrictions.
3/12/20	Events and gatherings with 500 or more people postponed or canceled.
3/12/20	Businesses with less than 500 individuals in attendance are required to cut capacity to 50 percent (exceptions include schools, hospitals, public buildings, mass transit, grocery stores, and retail stores).
3/12/20	Only medically necessary visits will be allowed at nursing homes.
3/12/20	Health screenings required for all nursing home workers each day when they enter a facility and require them to wear surgical masks to guard against asymptomatic spread.
3/12/20	SUNY Albany cancels all in-person classes for the rest of the semester.
3/13/20	The first public drive-through testing site in New Rochelle opens.
3/13/20	FDA gives NY State authority to conduct all COVID-19 testing at public and private labs.
3/14/20	Department of financial services will require insurance companies to waive copayments for telehealth visits.
3/16/20	Governor Cuomo issues executive order to increase hospital capacity.
3/16/20	Department of Financial Services announced a special enrollment period for uninsured New Yorkers.
3/17/20	Governor Cuomo announces a three-way agreement with the legislature on paid sick leave bill to provide immediate assistance for New Yorkers impacted by COVID-19.
3/20/20	Temporary closure of barber shops, nail and hair salons and related personal care services.
3/20/20	New York State on PAUSE executive order signed.
3/20/20	100% closure of non-essential businesses statewide.

Date	New York State Response to COVID-19
3/20/20	Matilda's Law enacted. People 70 years old and older and those with compromised immune systems and illness required to stay home and limit home visitations by others.
3/20/20	90-day moratorium on any residential or commercial evictions.
3/27/20	First 1000 bed temporary hospital at Jacob K Javits Convention Center.
3/29/20	NYS on PAUSE restrictions extended 2 weeks.
4/6/20	NYS on PAUSE restrictions extended 2 weeks.
4/12/20	Governor Cuomo executive order directs employers to provide masks for employees who interact with the public.
4/15/20	Governor Cuomo issues an executive order requiring all people in New York to wear masks or face coverings in public.
4/16/20	NYS on PAUSE restrictions extended until May 15.
4/17/20	Executive order directing all NYS public and private labs to coordinate with state DOH to prioritize diagnostic testing.
4/21/20	Elective outpatient surgeries may resume in low-risk areas.
5/9/20	Initiative to expand access to testing in Low-income communities and communities of color.
6/25/20	Counties to receive 323$ million from enhanced Medicaid funds in response to COVID-19.
