# Supplementary material for: Early COVID-19 Interventions Failed to Replicate 1918 St. Louis vs. Philadelphia Outcomes in the United States
Source: Front Public Health. 2020 Sep 15;8:579559. doi: 10.3389/fpubh.2020.579559 (PMC7522277; doi:10.3389/fpubh.2020.579559)
Supplement: Supplementary file 3 [file Table_3.PDF]

**Supplemental Table 3.** California state-level public health response to COVID-19 pandemic.

| Date | California State Response to COVID-19 |
|---|---|
| 1/26/20 | Two confirmed cases of coronavirus in California, 1 in Los Angeles County, 1 in Orange County. |
| 1/31/20 | Three confirmed cases of coronavirus, one new case in Santa Clara County. |
| 2/6/20 | CDPH and network of labs prepared to begin coronavirus testing in California. |
| 2/26/20 | CDC confirmed the first instance of coronavirus community transmission in California. |
| 2/28/20 | COVID-19 testing kits arrived at state public health laboratories. |
| 3/2/20 | State health & emergency officials ramped up response. Governor Newsom activated the State Operations Center (SOC) in Mather, California, to its second-highest level. |
| 3/3/20 | Governor Newsom, state health & emergency officials, announced the release of millions of N95 filtering facepiece masks for use in low-risk health settings to address shortages caused by COVID-19. |
| 3/4/20 | State declares State of Emergency. First known coronavirus death in Placer County, California. |
| 3/5/20 | Governor Newsom announced more than 22 million Californians now eligible for free medically necessary COVID-19 testing. |
| 3/7/20 | California released updated guidance for schools, colleges & large public events to prepare and protect Californians from COVID-19. |
| 3/9/20 | State health & emergency officials encouraged individuals at higher risk of severe illness due to COVID-19 to take precautions. |
| 3/11/20 | State health & emergency officials released guidance to prepare and protect homeless Californians and service providers from COVID-19. |
| 3/12/20 | Cancellation of large gatherings 250 or more. Social distancing (6ft), individuals at higher risk should not go to gatherings of 10 or more. |
| 3/13/20 | Guidance released to prevent the transmission of COVID-19 in gambling venues, theme parks and theaters. |
| 3/13/20 | Executive order mandates that school districts use dollars to fund distance learning and high-quality educational opportunities, safely provide school meals, and arrange for the supervision of students during school hours. |
| 3/16/20 | Seniors and COVID-19 vulnerable residents directed to home isolate. Governor Newsom issues Executive Order redirecting California agencies to protect licensed facilities, staff & residents most vulnerable to COVID-19. |
| 3/16/20 | Guidance released to prevent the transmission of COVID-19 in food and beverage venues. |

| Date | California State Response to COVID-19 |
|---|---|
| 3/16/20 | Executive order authorized local governments to halt evictions, slows foreclosures, and protects against utility shut offs. |
| 3/18/20 | Executive order to protect ongoing safety net services for most vulnerable Californians during COVID-19 outbreak. |
| 3/18/20 | Executive order made to waive, pending federal approval, this year's statewide testing for California's more than 6 million students. |
| 3/18/20 | Governor Newsom takes emergency actions & authorizes $150 million in funding to protect homeless Californians from COVID-19. |
| 3/19/20 | Stay at Home order, except for essential needs. |
| 3/20/20 | Executive order to permit vote-by-mail procedures to be used in three upcoming special elections, protecting public health and safety during the COVID-19 outbreak. |
| 3/21/20 | Order expands capacity to combat COVID-19 in health care facilities. |
| 3/24/20 | Executive order on state prisons and juvenile facilities. No new commitments to state prisons or juvenile facilities will be accepted for the next 30 days Order also directs videoconferencing of all scheduled parole suitability hearings starting next month. |
| 3/27/20 | Executive action authorizing local governments to halt evictions extended. |
| 3/27/20 | Executive order granting emergency authority to judicial council to be able to conduct business during COVID-19. |
| 3/30/20 | Executive order to expand health care workforce and staff at least an additional 50,000 hospital beds needed for the COVID-19 surge. |
| 3/30/20 | Order provides 90-day extension in state and local taxes, including sales tax order extends licensing deadlines and requirements for a number of industries. |
| 4/1/20 | Executive order that allows for the immediate use of funds to support the state's continuing efforts. |
| 4/1/20 | Guidance released on the use of cloth face coverings. |
| 4/2/20 | Executive order that will restrict water shutoffs to homes and small businesses while the state responds to the COVID-19. |
| 4/3/20 | Executive order comes in response to COVID-19 pandemic to limit price increases from sellers on critical items, such as food and medical supplies. |
| 4/3/20 | Executive order allows health care providers to use video chats and applications to provide health services without risk of penalty. |

| Date | California State Response to COVID-19 |
|---|---|
| 4/4/20 | Executive order to provide expanded access to childcare for essential workers during COVID-19 response. |
| 4/7/20 | Executive order that provides additional support for older adults and vulnerable young children. |
| 4/7/20 | Executive order to help the state procure necessary medical supplies to fight COVID-19. |
| 4/8/20 | Latest COVID-19 facts, including new data on racial demographics and expanded health care worker data announced. |
| 4/14/20 | Executive order addressed the release and reentry process at the Division of Juvenile Justice (DJJ) in response to the COVID-19 pandemic, so that eligible youth serving time at DJJ can be discharged safely. |
| 4/15/20 | Expansion of call center hours at the Employment Development Department (EDD) to better assist Californians with unemployment insurance applications EDD will also implement a one-stop shop for those applying for Pandemic Unemployment. |
| 4/16/20 | Executive order will benefit workers in grocery stores and fast-food chains and delivery drivers. Order will give two weeks of supplemental paid sick leave to certain food sector workers if they are subject to a quarantine or isolation order or medical treatment. |
| 4/17/20 | Executive order that allows for temporary waivers to certain foster youth programs to ensure continuity of care in response to the COVID-19 pandemic. |
| 4/23/20 | Governor Newsom announced a deal to expand student loan relief for 1.1. million Californians Governor also signs an executive order to stop debt collectors from garnishing individual COVID-19-related financial assistance. |
| 4/23/20 | Executive order empowered schools to focus on COVID-19 response and transparency. |
| 4/23/20 | Executive order on actions taken in response to COVID-19. 60-day extension for several DMV related issues. |
| 5/1/20 | Executive order that temporarily broadened the capability of counties to enroll persons into the California Work Opportunity and Responsibility to Kids (CalWORKs) program. |
| 5/6/20 | State launches California COVID-19 testing sites website. |
| 5/12/20 | California Department of Public Health announces new sectors that can reopen with modifications statewide. |
| 5/22/20 | California Connected, contact tracing program, and public awareness campaign launched. |
| 5/25/20 | Counties statewide can reopen places of worship for religious services and retail stores. |
| 5/26/20 | Most counties can reopen barbershops and hair salons with modifications. |

| Date | California State Response to COVID-19 |
| --- | --- |
| 6/18/20 | Californians now required to wear a mask in most settings outside the home. |
| 6/28/20 | Governor acts to close bars in specific counties. |
