# Supplementary material for: Early COVID-19 Interventions Failed to Replicate 1918 St. Louis vs. Philadelphia Outcomes in the United States
Source: Front Public Health. 2020 Sep 15;8:579559. doi: 10.3389/fpubh.2020.579559 (PMC7522277; doi:10.3389/fpubh.2020.579559)
Supplement: Supplementary file 4 [file Table_4.PDF]

Supplemental Table 4. Florida state-level public health response to COVID-19 pandemic.

Date	Florida State Response to COVID-19
3/1/20	Department of Health announces two presumptive positive cases of COVID-19 in Florida.
3/6/20	Two confirmed COVID-19 deaths.
3/7/20	Department of Health advises individuals who traveled on Nile River cruise to self-isolate for 14 days from the date of return.
3/9/20	State of Emergency declared.
3/16/20	President Trump & CDC issue the "15 Days to Slow the Spread" guidance advising individuals to adopt far-reaching social distancing measures, such as working from home and avoiding gatherings of 10 or more people.
3/17/20	Restrictions placed on bars, pubs, nightclubs (Closed), restaurants (50% occupancy, 6ft distancing, employee screening) and beaches (no groups larger than 10, 6ft distances between parties, beach closures at the discretion of local authorities).
3/20/20	Broward and Palm Beach County beaches closed.
3/20/20	Restriction placed on alcohol sales for takeout and delivery suspended. Restaurants closing for dine in customers; take-out and delivery only. Gyms and fitness center closures.
3/20/20	Non-essential and elective surgeries/procedures are postponed or canceled in order to preserve essential equipment.
3/23/20	Executive order for airport screening and isolation directs all persons traveling to Florida from an area with substantial community spread to isolate or quarantine for a period of 14 days from the time of entry into the state.
3/24/20	Individuals must isolate after entering Florida from the Tristate region for 14 days.
3/24/20	Public health advisory for people 65 years old and older to stay home and to take other measures to limit their risk of exposure to COVID-19.
3/24/20	Public health advisory for individuals with serious underlying health issues.
3/24/20	Ban on gatherings of 10 or more people.
3/24/20	Public health advisory urging people who can work remotely to do so.
3/27/20	Individuals traveling to Florida from roadways must isolate for 14 days. Checkpoints present on roadways.
3/27/20	Vacation rentals and third-party platforms cannot take new reservations and suspend vacation rental operations.

Date	Florida State Response to COVID-19
3/30/20	Re-employment of essential personnel from retired status.
3/30/20	Miami-Dade County, Broward County, Palm Beach County, and Monroe County public access restrictions to essential businesses only.
3/31/20	Broward Country and Palm Beach County beaches remain closed.
4/1/20	Safer at home executive order for seniors and individuals with underlying illnesses until April 30th.
4/10/20	Vacation rental closures extended to April 30th.
4/29/20	Limited extension of essential services and activities and vacation rental prohibited until May 4th, 2020.
4/29/20	Florida reopens. “Phase 1: Safe. Smart. Step-by-Step.” Restaurants, museums, and in-store retail establishments may open and have 25% of capacity while practicing distancing. Elective surgeries may resume in low-risk areas.
5/5/20	State launches Community Action Survey “Stronger Than C-19.”
5/5/20	State launches COVID prevention page.
5/8/20	Executive order extends state emergency.
5/9/20	Reopening state expanded.
5/14/20	State-supported community-based testing sites to temporarily close due to a low-pressure system. Phase 1 reopening for Miami-Dade and Broward counties.
5/16/20	Miami-Dade County state-run community-based testing sites to reopen on Sunday.
5/22/20	Reopening of state expanded.
6/3/20	State of Florida announced three new COVID-19 testing sites through a partnership with Publix.
6/5/20	Florida Division of Emergency Management sends 500,000 gowns to long-term care facilities.
6/8/20	Home Depot partner to provide three COVID-19 testing sites.
6/27/20	The Florida DOH signs on to the U=U campaign.
