# Supplementary material for: Early COVID-19 Interventions Failed to Replicate 1918 St. Louis vs. Philadelphia Outcomes in the United States
Source: Front Public Health. 2020 Sep 15;8:579559. doi: 10.3389/fpubh.2020.579559 (PMC7522277; doi:10.3389/fpubh.2020.579559)
Supplement: Supplementary file 5 [file Table_5.PDF]

Supplemental Table 5. Texas state-level public health response to COVID-19 pandemic.

Date	Texas State Response to COVID-19
3/4/20	Department of State Health Services (DSHS) announced the first case of COVID-19 in Texas.
3/9/20	Texas' case of COVID-19 without international travel likely contracted from a recent trip to California.
3/11/20	COVID-19 case with no travel or known exposure identified.
3/15/20	DSHS distributed additional medical supplies for COVID-19 response.
3/17/20	First COVID-19 related death.
3/19/20	Texas health commissioner declares a public health disaster.
3/19/20	Executive order GA-08. Schools closed. Avoid gatherings of 10 or more people. No visiting nursing homes, retirement or long-term care facilities unless critical.
3/22/20	Postponement of all surgeries and procedures that are not immediately medically necessary.
3/24/20	Executive order GA-10 DSHS accelerated COVIOD-19 case reporting.
3/26/20	Executive order GA-11 Increased airport screening for individuals coming from the Tri State area, must self-quarantine for 14 days upon arrival.
3/29/20	Executive order GA-12 Roadway screening for individuals coming from Louisiana must self-quarantine for 14 days upon arrival.
3/29/20	Executive order GA-13. Detention of inmates and regulations on who may be released.
3/31/20	Executive order GA-14 extending prior recommendations essential businesses, social distancing, school closures, restaurants, etc. until April 30, 2020.
4/17/20	Executive order GA-15 postponement of elective surgeries and procedures extended until May 8, 2020.
4/17/20	Executive order GA-16 safe strategic opening of select services as a 1st step to opening Texas. Starting April 24, 2020, non-essential businesses that can-do delivery by mail, delivery to doorstep, or pickup may open.
4/17/20	Executive order GA- 17 establishment of COVID-19 Strike force, an advisory committee to reopening the state.
4/27/20	Executive order GA-18 expanded reopening of services, effective until May 15, 2020.
4/27/20	Executive order GA-19 beginning May 1, 2020, healthcare facilities may begin elective procedures/surgeries but must conserve 15% of resources for COVID-19 patients.
4/27/20	Executive order GA-20 rescinds GA-11 & 12 on travel and self-quarantining.

Date	Texas State Response to COVID-19
5/5/20	Executive order GA-21 expansion of reopened services.
5/7/20	Executive order GA-22 cosmetology salons allowed to reopen.
5/12/20	DSHS distributes Remdesivir to hospitals to treat COVID-19 patients.
5/18/20	Executive order GA-23 expansion of reopened services.
5/21/20	Executive order GA-24 termination of air travel restrictions.
5/22/20	Executive order GA-25 visitations reopened at the county and municipal jails.
6/3/20	Executive order GA-26 expansion of reopened services.
6/25/20	Executive over GA-27 need for increased hospital capacity- postponement of all surgeries and procedures that are not medically necessary.
6/26/20	Executive order GA -28 expansion of reopened services.
